# The Discriminant Power of Specific Physical Activity and Dietary Behaviors to Distinguish between Lean, Normal and Excessive Fat Groups in Late Adolescents

**DOI:** 10.3390/nu15051230

**Published:** 2023-02-28

**Authors:** Jarosław Domaradzki

**Affiliations:** Department of Biostructure, Wroclaw University of Health and Sport Sciences, 51-612 Wrocław, Poland; jaroslaw.domaradzki@awf.wroc.pl

**Keywords:** body fat percentage, adolescents, physical activity intensity, dietary behaviors, discriminant analysis, discriminating set of variables

## Abstract

Physical activity (PA) and dietary behaviors (DBs) are crucial determinants of body mass composition. This work is a continuation of the previous study of PA and DBs patterns in late adolescents. The main aim of this work was to assess the discriminant power of PA and dietary behaviors and to identify the set of variables that discriminated participants with low, normal, and excessive fat the most. The results were also canonical classification functions that can allow the classification of individuals into adequate groups. A total of 107 individuals (48.6% male) participated in examinations, which used the International Physical Activity Questionnaire (IPAQ) and Questionnaire of Eating Behaviors (QEB) to assess PA and DBs. The participants self-reported body height, body weight, and BFP, with the accuracy of the data confirmed and empirically verified. Analyses included the metabolic equivalent task (MET) minutes of PA domains and intensity, and indices of healthy and non-healthy DBs, calculated as a sum of the intake frequency of specific food items. At the beginning, Pearson’s r-coefficients and chi-squared tests were calculated to study various relationships between variables, while the main considerations were based on discriminant analyses conducted to determine the set of variables with the most power to distinguish between lean, normal, and excessive body fat groups of participants. Results showed weak relationships between PA domains and strong relationships between PA intensity, sitting time, and DBs. Vigorous and moderate PA intensity related positively to healthy behaviors (r = 0.14, r = 0.27, *p* < 0.05), while sitting time related negatively to unhealthy DBs (r = −0.16). Sankey diagrams illustrated that lean persons displayed healthy DBs and low sitting time, while those with excessive fat had non-healthy DBs spent more time sitting. The variables that effectively distinguished between the groups include *active transport* and *leisure time* domains alongside low-intensity PA, represented by *walking intensity* and *healthy dietary behaviors*. The first three variables participated significantly in the optimal discriminant subset (*p* = 0.002, *p* = 0.010, *p* = 0.01, respectively). The discriminant power of the optimal subset (contained four above-mentioned variables) was average (Wilk’s Λ = 0.755) and determined that weak relationships between PA domains and DBs resulted from heterogeneous behaviors and mixed patterns of behaviors. Identifying the trajectory of the frequency flow through specific PA and DBs allowed for well-designed tailored intervention programs to improve healthy habits in adolescents. Therefore, identifying the set of variables that discriminate the most between lean, normal, and excessive fat groups is a suitable target for intervention. The practical achievements are canonical classification functions that can be used to classify (predict) participants in groups based on the three the most discriminating PA and DB variables.

## 1. Introduction

Excessive body fat results from several factors, which include weak physical activity (PA), a sedentary lifestyle, and poor dietary behaviors (DBs). Obesity is still growing in prevalence [1] and is considered a global pandemic [2], with evidence suggesting that the extent of the problem has tripled since 1970 [3]. The consequences of obesity on health are multidimensional and include several outcomes such as metabolic disorders, cardiovascular diseases, poor mental health, and deterioration of physical performance [4,5,6,7,8].

Many studies have focused on the relationship between PA and DBs [1,9,10,11], with most highlighting a mixture of healthy and unhealthy behaviors through assessing patterns of PA (or inactivity) and eating behaviors in young people [12,13]. Moreover, some studies demonstrated associations between specific PAs and food intake, suggesting a relationship between the time spent on PA and eating behaviors. In particular, increased consumption of healthy food items, such as fruits and vegetables and lower amounts of soft drinks, relates to higher levels of exercise [14,15].

PA can be considered through the prism of domains, intensity levels, and global nutrition indicators. However, a gap exists in the literature as no studies have examined the relationship between PA and DBs and their combined effects in body mass composition. In addition, studies attempting to indicate the variables most related to body mass composition, particularly lean, normal, and excessive body fat, are lacking. Furthermore, no studies have identified the PA and DB variables that discriminate the most between groups of adolescents with different levels of body fat. Despite many works showing PA and DB patterns using principal component analysis (PCA), approaches using analysis of variance (ANOVA) or regression models [16,17,18] are unclear on which variables have the most power in distinguishing between groups with differing body fat. Therefore, this study presents a novel discriminant analysis approach to exploring the relationships between PA and DBs.

Control functions of PA and dietary habits used for regulating body weight as a whole, and body fat in particular, are well documented [19,20,21]. Some studies using the International Physical Activity Questionnaire (IPQA), for example, suggest that the PA task domains appear to be less effective than PA intensity. Indeed, moderate to vigorous PA (MVPA) provides more substantial health benefits than low-intensity PA [16,18]. Similarly, the results regarding the assessment of importance of healthy and unhealthy dietary behaviors for body mass composition are still unclear [16,17]. The additive influence and hierarchy of the strength of effect on body fat percentage (BFP) within sets of PA and dietary components remain unknown.

The most efficient intervention programs created for the fight against obesity combine PA and dietary recommendations [22,23]. However, lifestyle changes, particularly the increased consumption of food containing oils in late adolescents and increased use of sugar-sweetened beverages in college students [24,25], suggest a need to continue searching for variables related to BFP that can potentially distinguish between individuals with low and excessive levels of fat. Therefore, the main aim of this work was to assess the discriminant power of PA and dietary behaviors and to identify the set of variables that discriminated participants with low, normal, and excessive fat the most. The results were also canonical classification functions, which can allow to classify individuals to adequate groups.

## 2. Materials and Methods

This work constitutes the second part of a study on the effects of PA and DBs on body mass composition in late adolescents. A detailed description of the sample size estimation, study design, participant recruitment, data collection, data handling, input of missing data, validation, and assessment of the consistency between self-reported and empirically measured body weight and BFP, as well as the Yeo–Johnson power transformation of data to a normal distribution, were published previously [13]. Herein, a summary of the data is given.

### 2.1. Ethics

The Research Bioethics Committee of the Faculty Senate of the Wroclaw University of Health and Sport Sciences approved the study (consent numbers 33/2018 and 13/2022). The study followed the ethical principles for medical research involving human subjects contained in the Declaration of Helsinki published by the World Medical Association.

### 2.2. Participants and Study Design

Power analysis conducted for clustering procedures, presented in the first part of the study, indicated a requirement for a minimum of 60 males and 60 females. However, the final analysis included 107 participants.

**Figure 1:**
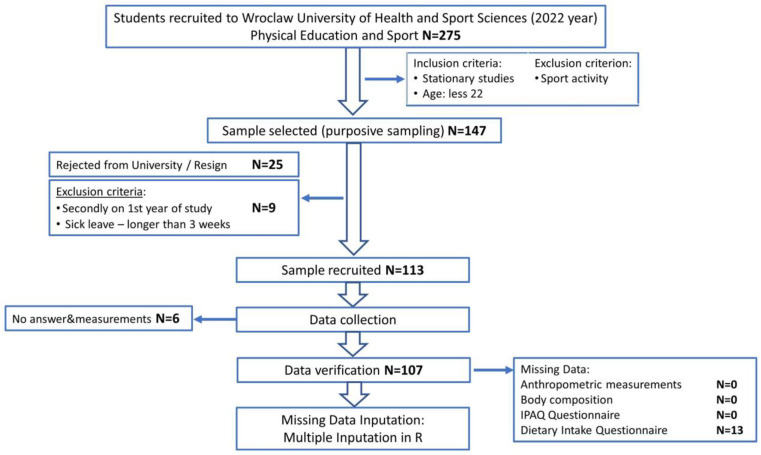
Flowchart of study design and data collection.

Participants included 107 healthy individuals (52 males (48.6%)) recruited in 2022 from students in their 1st year of study at the Faculty of Physical Education and Sport at Wroclaw University of Health and Sport Sciences. A flowchart describes the sampling procedure (Figure 1).

Of 275 students recruited to the university in 2022, 147 took part in the examinations. After eliminating students rejected from the university (*n* = 25), those who met exclusion criteria (*n* = 9), and those who did not answer the questionnaires (*n* = 6), 107 participants took part in the study.

### 2.3. Data Collection and Measurements

Data collection utilized the local Student Health Behaviors Studies (STUHB22) project, which evaluates PA, DBs, attitudes towards health, lifestyle, intrinsic and extrinsic risk factors for injuries during PA, and risk factors for overweight and obesity.

Sports field studies students completed online questionnaires using Google Forms immediately after an academic lecture (Human Anatomy taught by the author) during the 2022 academic year. The author of this article conducted recruitment, data collection, and data entry. The study used the Polish version of the International Physical Activity Questionnaire (IPAQ) (long version) [26] and the Questionnaire of Eating Behaviors (QEB) [27]. IPAQ showed moderate validity and was similar to studies when compared to accelerometers (r ≈ 0.4), while QEB showed great internal reliability (Fleiss’ kappa: 0.64–0.84). The analysis also included self-reported body height, weight, and BFP data. In addition, the R sample function randomly selected a subset of 19 participants, including nine males and ten females, from an alphabetical list for manual anthropometric measurement. In this work, four domains and three intensity levels of PA derived from IPAQ were considered. Domains were: work/school—PA related to activity in work or/and school; active transport—PA related to the way of commuting during the day (on foot, cycling, or using private or public transportation); domestic/gardening—PA related to housework and yard activities; leisure time—PA related to activities during leisure time. Intensity levels were: vigorous, moderate, and walking. Activities during the last 7 days were considered. The physical activity level was expressed as a standard Metabolic Energy Turnover (MET) in METminutes/week [26]. Calculations were based on corresponding MET values assigned to various activities: walking = 3.3, moderate = 4.0, cycling = 6.0, and vigorous = 8.0. The result for each participant was METminutes/week scores computed by multiplying the MET value by the time spent on these activities. In addition, sitting time, as an inactivity domain, was collected. The minimal set of 16 questions recommended in the instruction of the QEB was used in this work [27]. Two indices on diet quality (modules) were calculated into a healthy dietary habits index (8 items: whole-grain bread, milk, fermented milk drinks, curd cheese (including homogenized cheese), fish and fish dishes, bean and pea dishes, fruits, and vegetables) and an unhealthy dietary habits index (fast food, fried foods, cheese (including cream cheese), sweets, confectionery, canned meat, canned fish or canned vegetable-meat, sweetened carbonated beverages, energy drinks, and alcoholic drinks).

Anthropometrical and body composition measurements used standard procedures and included two body height measurements with an accuracy of 0.1 cm using an anthropometer (GPM Anthropological Instruments, DKSH Ltd., Zürich, Switzerland). A bioelectric impedance method assessed body weight and BFP using an InBody230 device (InBody Co., Ltd., Cerritos, CA, USA).

A statistical approach assessed the reliability of the self-reported BFP and body weights used in the analyses. A comparison of the slopes and intercepts of simple regression analyses of the empirical and self-reported data confirmed the reliability of the latter [28].

Although there were no missing anthropometrical, body composition, or PA data, there were missing data for the QEB questionnaire (*n* = 13). The propensity for a data point to be missing was random, also known as missing completely at random (MCAR) [29,30]. All measurements were preprocessed by applying multiple imputations in the R language using the software RStudio v.2022.7.1.554 (RStudio Team (2022). RStudio: Integrated Development Environment for R. RStudio, PBC, Boston, MA, USA URL http://www.rstudio.com/ (accessed on 15 November 2022)) with the *mice* package (v.3.14.0).

### 2.4. Statistics

The Shapiro–Wilk test evaluated the normality of data distribution, with the variables identified as non-normally distributed transformed to a normal distribution shape using the Yeo–Johnson power transformation [31]. Transforming the data to bring them closer to normality was required to meet the assumptions of the statistical methods, with all variables standardized to mean = 0 and standard deviation (SD) = 1 for each sex. This procedure allowed for the analysis of the whole group without dividing them by gender. Continuous variables were presented as means and 95% confidence intervals (CI), while categorical variables were presented as numbers and percentages.

The procedures of a discriminant analysis need knowledge about simple associations between variables and relations between subgroups. Therefore, as a first stage of analysis, Pearson’s product–moment correlation coefficients were calculated. Graphically correlation matrices were presented in chord diagrams using the *circlize* package in R [32]. Chord diagrams are a specific type of flow diagram that illustrate relationships between variables, with the width of the link being proportional to the strength of associations. Chord diagrams can be particularly useful for displaying inter-relationships between two or more groups of items, whereas the numbers of individuals in various subgroups, such as those preferring specific PA domains or healthy DBs, were presented as percentages, with quantiles used to represent sitting time. The interrelationship between PA domains, PA intensity, sitting time, DBs, and the corresponding BMI, BFP, and FMI categories, was assessed based on the prevalence of participants and their choice of PA and DBs. The preferred behavior was considered to be the one for which participants scored the most points. Differences in proportions between various subgroups were tested with the chi-squared test (χ^2^). Graphically, trajectories of the number of males and females flowing from PA domains or intensity, through sitting quintiles, and DBs, to body composition categories, were presented as Sankey diagrams. Sankey diagrams are not a statistical method, but visually depict a flow from one set of values to another. In this work, Sankey diagrams present the interrelationships between different subcategories of participants. The graphs were prepared in Google Sheets using the ChartExpo add-on.

BFP used in Sankey diagrams was categorized according to the American Journal of Clinical Nutrition, which states that males should have 8–19% body fat and females should have 21–32%. A BFP of less than 8% in males is considered a fat deficit, with 8–14% considered lower normal (1st), 14–19% upper normal (2nd), and above 19% considered excessive fat. Meanwhile, less than 21% body fat is a deficit in females, with 21–27% considered lower normal (1st), 27–32% upper normal (2nd), and above 32% considered excessive.

The main approach was based on discriminant analysis (DA). This method allows for identifying the set of variables that distinguish between the four BFP categories the most, which, unlike analysis of variance, allows for multivariate analyses. Discriminant analysis procedures allow for building canonical discriminant functions that are the linear combination of independent variables that will discriminate between the categories (e.g., lean, normal and excessive fat groups of participants) in a perfect manner [28]. DA also allows one to determine the classification functions that can be used to classify participants into specific groups. It is a practical application of the DA. Discriminant analysis with the best subsets selection of predictor effects was conducted, with Wilks’ Lambda value set as the criterion for choosing the best subset of predictor effects, and searching of the four-element subsets was ordered. Wilk’s Lambda is the primary statistic usually used to assess the discriminant power of the subset of the identified variables, whereas the ANOVA tests were used to test the significance of every single variable in subset. Tolerance values were calculated as a 1-R^2^ for each variable in relation to the rest of the variables. Tolerance is the proportion of a variable’s variance not accounted for by other independent variables in the equation. Raw and standardized coefficients (*β*) of the canonical discriminant functions were calculated. Raw values are used to build discriminant equations, while *β–values* allow for a comparison of the discriminant power between variables. Coefficients of the classification functions were calculated in addition. These can be used to predict subjects’ group membership. At the end, hierarchical cluster analysis (HCA) assessed the similarities and dissimilarities between BFP categories, with Mahalanobis distances and the Ward method of linkage used to agglomerate the set of variables that best discriminated between groups.

All statistical tests and procedures used a *p*-value equal to 0.05 to determine the significance level, and all calculations (except those using RStudio) employed Statistica 13.0 (StatSoft Poland 2018, Cracow, Poland).

## 3. Results

The basic characteristics of the males and females were presented previously [13]. Sex differences in the active transport domain and non-healthy dietary behaviors revealed that males had significantly lower mean values than females (*p* = 0.027 and *p* = 0.036, respectively). In addition, a lower value for total sitting time in males was very close to significance (*p* = 0.076). Comparing PA and DB patterns, several similarities and dissimilarities emerged between both sexes. One of the common phenomena included the relationship between some unhealthy PA and DBs (low-intensity PA, longer sitting time, and poor food consumption). Moreover, the preliminary analysis suggested a possible combined effect of such behaviors on body composition. Therefore, this work analyzed the multivariate relationship between PA and dietary behaviors and their impact on body fat percentage in-depth.

The analysis in this work was divided into two parts. The first stage for discriminant analysis was to study the interrelationship between PA domains, sitting time, and dietary behaviors. The approach was based on simple Pearson correlations and χ^2^ tests. Results were illustrated with chord and Sankey diagrams. The second stage, as a main analysis, was descriptive discriminant analysis conducted in order to identify a set of variables.

### 3.1. The Interrelationship between Physical Activity Domains/Intensity, Sitting Time, and Dietary Behaviors

**Figure 2:**
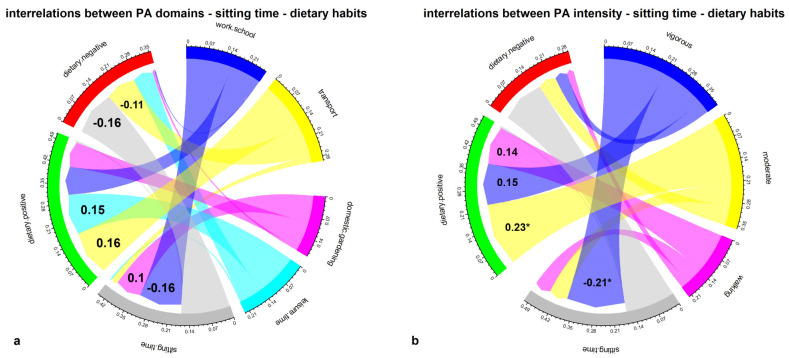
(**a**,**b**) Chord diagrams of the Pearson correlations between: (**a**) PA domains, sitting time, and dietary healthy (positive) and unhealthy (negative) behaviors in adolescents; (**b**) PA intensity, sitting time, and dietary healthy (positive) and unhealthy (negative) behaviors in adolescents. The width of the links is proportional to the strength of the correlation. * *p* < 0.05.

**Figure 3:**
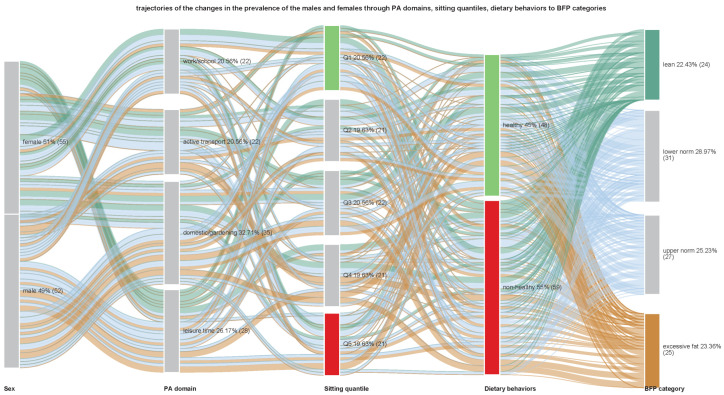
Sankey diagram of the trajectories of the relationship between dominant physical activity domains, sitting time quintiles, and healthy and unhealthy dietary behaviors, with corresponding body fat percentage categories, based on the prevalence of the participants. The width of the links is proportional to the number of individuals.

**Figure 4:**
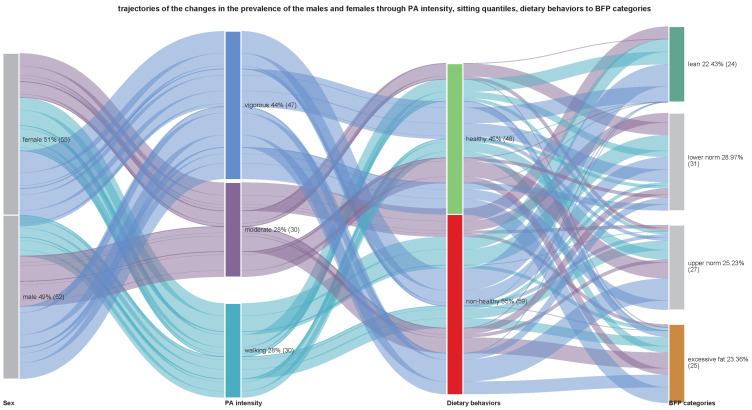
Sankey diagram of the relationship trajectories between dominant physical activity intensity and healthy and unhealthy dietary behaviors based on the prevalence of participants in body fat percentage categories. The width of the links is proportional to the number of individuals.

**Figure 5:**
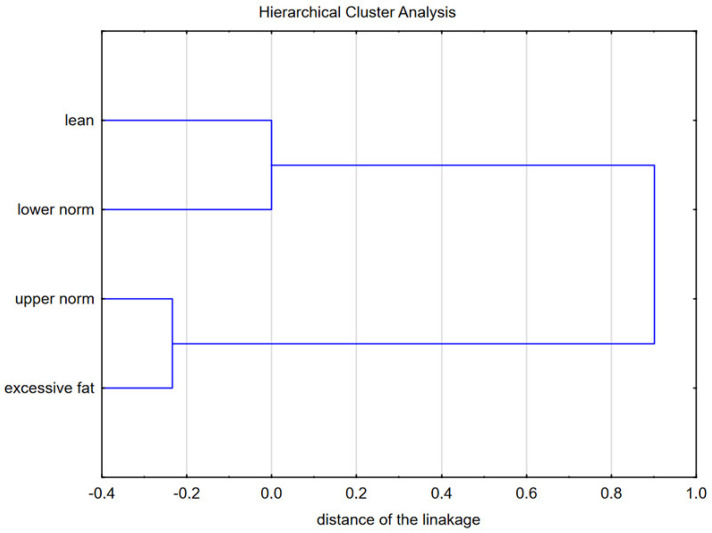
Cluster analysis of the groups of lean, lower normal, upper normal, and excessive fat participants based on the best-discriminating subset of variables (active transport, leisure time, walking intensity, and healthy dietary behaviors).

Initial examinations included the interrelationships between PA domains and PA intensity, with sitting time and dietary behaviors, by calculating Pearson-product correlation matrices and producing chord diagrams (Figure 2 and Figure 3). Meanwhile, Sankey diagrams summarized the relationship between the prevalence of the participant’s preference for PA domains or PA intensity concerning sitting time, healthy DBs, unhealthy DBs, and corresponding BFP categories (Figure 4 and Figure 5). These mainly visual presentations of the relationships were the starting point in designing discriminant analysis and multivariate multiple linear regression models.

Figure 2a shows the correlations between dominant PA domains, sitting time, and DBs. PA related to the work/school and domestic/gardening domains relate to sitting time compared to active transport and leisure time. However, none of those correlations were statistically significant (*p* > 0.05). On the other hand, sitting time was negatively related to unhealthy DBs (r = −0.16), which suggests a minor relationship between time spent sitting and the frequency of eating unhealthy foods. PA leading to leisure time and active transport are the domains most related to healthy eating behaviors (r = 0.14 and r = 0.16, respectively), indicating that more PA supports healthy behaviors.

Figure 2b shows the correlations between dominant PA intensity domains, sitting time, and DBs. Vigorous-intensity PA is mainly related to healthy DBs (r = 0.14), although this relationship was not significant. Thus, there was a significant relationship observed for moderate-intensity PA (r = 0.23, *p* < 0.05), while walking (low intensity) was also positively related (r = 0.14). The strongest, negative, and significant relationship with sitting time presented PA vigorous intensity (r = −0.21, *p* < 0.05).

Figure 3 shows the trajectory of the changes in the prevalence of the participants in relation to dominating PA domains, sitting time quantiles and dietary behaviors in relation to the body fat percentage categories. As it is seen, in females, dominating PA domains are active transport and domestic/gardening domains. While in males it is domestic/gardening and leisure time domains. More interesting information can be seen by reading the diagram from the body fat categories. Opposite categories are marked in color. Lean persons (62%—both males and females) prefer healthy dietary behaviors and excessive fat persons (44%). The difference, however, was not statistically significant (χ2 = 1.07, *p* = 0.785) with small non-healthy behaviors. Healthy behaviors are related to lower sitting time (41%—quantiles Q1 and Q2), while negative behaviors mostly to higher sitting time (44%—quantiles Q4 and Q5). However, differences in proportions were not significant (χ2 = 2.64, *p* = 0.620). Persons with excessive fat, to a large degree, prefer non-healthy dietary behaviors, and they are related to more time spent sitting. On the other hand, tracking yellow lines leading from sex to excessive fat persons, as well as green lines leading to lean persons, showed that more males are included in excessive fat categories, while females mostly agglomerated in lean categories. Differences were statistically significant (χ2 = 35.28, *p* < 0.001).

Figure 4 shows the trajectory of changes in the prevalence of participants in relation to dominant PA intensity and DBs with BFP categories. PA intensity flow demonstrated similar proportions in both sexes for vigorous and moderate walking intensity. Interestingly, more individuals with less intense PA related to non-healthy DBs (56.7%) and the upper normal and excessive fat categories. In contrast, healthy dietary behaviors related to vigorous-intensity PA in most participants (both males and females (46.8%). However, the differences in proportions were not statistically significant (χ^2^ = 0.13, *p* = 0.938). Indeed, most participants with excessive fat preferred non-healthy eating (56.0%) and moderate-intensity PA. (42.9%). Differences in proportions were not significant (χ^2^ = 3.24, *p* = 0.198).

### 3.2. The Set of Physical Activity and Dietary Behaviors That Effectively Distinguished between Lean, Normal, and Excessive Fat Groups

Based on the results so far, it was reasonable to look for a set of PA and DB variables that differentiate individuals with different levels of adiposity the most.

Standardized mean values with 95% CI are presented in Table 1. The results of the discriminant analysis are presented in Table 2.

The results of discriminant analysis are limited to the indication of the set of variables that most discriminate between the four groups of participants. In-depth analysis of the discriminating functions separating groups of participants, their eigenvalues, structural coefficients, and predicted classifications were omitted. The set of variables best differentiating between the groups contained *active transport* and *leisure time* domains, low-intensity PA (*walking intensity*), and *healthy dietary behaviors*.

Table 2 presents the calculated tolerances, which are 1-R^2^ for each variable in relation to the rest of the variables. Tolerance is the proportion of a variable’s variance not accounted for by other independent variables in the equation. All three PA domains were statistically significant, while dietary behaviors were not. All four variables explained 25% of the between-group variance, which confirmed Wilks’ lambda (Wilk’s Λ = 0.755). The first three variables were found in all earlier steps of analysis, indicating their high discriminatory power. Nonetheless, healthy DBs replaced unhealthy behaviors in the second last step and the vigorous PA domain in the previous step. The first and the second discriminant functions (eigenvalues: 0.232, 0.088, respectively), which separated groups in the best way, explained overall 89% of the between-groups variance. The raw coefficients of the functions presented in Table 3 can be used to construct equations to separate participants based on four discriminant variables. The load of each variable to the overall discriminant power of the whole optimal subset was assessed based on standardized () coefficients (Table 3). As it is seen, the greatest load to the first function was active transport (*β* =−1.15) and low intensity represented by the walking domain (*β* = 1.42). The second function is determined mainly by leisure time (*β* = 0.91). All three PA variables occurred more diagnostic than dietary healthy behaviors. The first function separates mainly participants who are active commuters from those who use privet or public transport, while the second function distinguishes between physically active and non-active during leisure time. ANOVA one-way tests for each variable confirmed the highest and statistically significant discriminant power of the active transport (F = 5.49, *p* = 0.002) and the lowest and nonsignificant (but quite close to significance) of the healthy dietary behaviors (F = 2.17, *p* = 0.095).

Based on the best subset four canonical classification functions were calculated, and four classification equations are presented in Table 4. It can be used to construct the equation formulas to classify participants into the adequate BFP subgroup. The example equation for the prediction of the individuals to lean group (C_lean_) can be written as:

C_lean_ = −1.80-at + 0.27 × lt + 1.13 × walk − 0.59 × hdi,

where C_lean_—1st classification function; at—PA value (MET-min/week) from active transport domain; lt—PA value (MET-min/week) from the leisure time domain; walk—PA value (MET-min/week) from walking domain; hdi—scores from healthy dietary behaviors.

Moreover, HCA allowed for a visual assessment of the similarities between the four groups of participants based on the four variables in the best-discriminating set, the results of which are presented in Figure 5. As expected, persons with lean and lower normal body fat had more similar behaviors than people with higher fatness (upper normal and excessive fat, which were similar to each other).

## 4. Discussion

This work mainly aimed to identify the set of variables that most discriminated between participants separated into subgroups based on BFP. In addition, an initial part of the main analysis was to assess interrelations between PA domains, intensity, time spent sitting, and healthy and non-healthy DBs. The study demonstrated a minor relationship between PA domains, sitting time, and DBs. On the contrary, stronger associations were observed for PA intensity and DB. Sankey diagrams provided a well-illustrated trajectory of the frequency flow of participants through specific PA and DB areas and clearly showed that the participants with excessive fat displayed non-healthy DBs and spent more time sitting. Individuals with a lean body mass preferred vigorous-intensity PA, while persons with excessive body fat preferred low or moderate-intensity PA. The main achievement, received with discriminant analysis, was to identify the set of four variables that most effectively distinguished between lean, normal, and excessive fat groups of participants. The set contained *active transport*, *leisure time*, low-intensity PA represented by *walking intensity*, and *healthy dietary behaviors*. As a practical result, the canonical discriminant functions were determined. They can be used for classification of individuals into groups with lean, normal, and excessive fat mass.

The initial stage of analysis was to assess the associations between PA and DB variables and the relationship between subgroups of participants preferring specific PA and DB domains. It occurred that healthy DBs increased modestly in proportion to increased leisure time and active transport. Moreover, an even stronger relationship was observed between healthy DBs and vigorous-intensity PA. Meanwhile, sitting time was related negatively to unhealthy DBs. These results are in agreement with Mitchell et al. [33], who studied associations between PA and other health behaviors during the coronavirus disease (COVID-19) pandemic. The authors observed modest improvements in nutritional behaviors in parallel with an increase in PA. This small, but noticeable, link appears to be a common observation after the lockdown experienced globally [34,35,36]. Furthermore, these results are in line with pre-pandemic studies showing that physically active persons preferred the consumption of healthier food (fruits, vegetables, lower fat savory foods, and water), while less active or non-active people preferred non-healthy foods (meat, fast food, sweetened beverages, or energy drinks) [37,38]. The mechanism that inhibits the need for calorific food is linked to reduced ghrelin levels (the hormone responsible for appetite), due to the acute effects of exercise [39,40]. The increase in non-healthy foods in the diet of physically inactive persons, including adolescents, can be related to common changes in dietary habits and the exchange of good food with poor food, especially fast-food. The results of Pearson’s correlations were in line with the analysis of the frequencies of participants’ preferred PA (called PA domains) and PA intensity in relation to their BFP classification by tracking changes in those frequencies. Using diagrams to visualize time spent sitting, healthy DBs, and non-healthy DBs made it possible to assess their role. Focusing on PA intensity and considering both sexes, males preferred vigorous and moderate intensity slightly more often, while females preferred moderate and low intensity. However, there were no differences in PA domain preferences, except for leisure time. Moreover, most persons preferring vigorous PA spent less time sitting and had healthy DBs. Regarding sitting time, there were no differences when comparing the numbers of less active participants, which was surprising but was also noted in previous literature [41,42].

More persons with healthy DBs were moderately or vigorously physically active, with less than 30% preferring low-intensity PA. This is in line with observations before the pandemic, which suggested that 27.5% of young adults do not achieve the levels of PA necessary for health [43]. In addition, other studies indicated that those who were not active preferred non-healthy food, which is in line with the frequency analysis in this study.

The main stage of analysis was to identify the set of variables that might efficiently discriminate participants between subgroups previously separated based on body fat percentage (namely lean, normal, and excessive fat). This kind of analysis, which is a novel approach to the problem, has two main benefits. The first is a set of variables identified as the most discriminated subgroups. This set can be treated as variables worth targeting to prevent overweight (i.e., stimulating PA domains and shaping attitudes related to dietary behaviors). The second one is the determination of canonical discriminatory functions for classifying individuals into groups differing in BFP. To the authors’ best knowledge, no works in the field of PA and DB relationships have attempted to solve the aforementioned problem. Most analyses aimed at assessing the variability between groups of subjects with different PA levels, body composition, or DBs used ANOVA or various regressions. This approach made it possible to highlight individual variables for which groups significantly differed and identify predictors of dependent variables. The multidimensional approach indicated the four-element subset that best differentiates the selected groups. The subset contained *active transport*, *leisure time*, *walking intensity* (lowintensity), and *healthy dietary behaviors*. HCA of the groups with different BFP confirmed similarities between lean and lower normal groups and between upper norm and excessive fat groups, separately, as well as dissimilarities between these clusters.

Participants (both male and female) who are active commuters, active during leisure time even with low-intensity and healthy eating were more likely lean or have normal body fat than participants who are not related to these domains. The coexisting components found in this study are partially in line with other authors. Most studies have shown a beneficial effect of leisure time on body weight and body fat mass, which was confirmed in systematic reviews [44,45]. Leisure time occurred as the most important variable (0.91, *p* < 0.05). This is convergent with results suggesting significant risk factor for being overweight and obese included no frequent leisure time physical activity [46]. However, contrary to these studies two facts: the level of intensity of the PA in leisure time and the prevalence of overweight participants who are not active during leisure time, but in relation to sex. Our own results showed low-intensity PA as a one of the key variables differentiating the groups, while Winkvist et al., showed moderate and vigorous intensity as more significant. Apart from that, own results showed also more overweight, no-active females than males, while Winkvist et al., showed opposite proportions (9.9% vs. 15.1%, respectively). The explanation for the difference could be the age of the participants. Winkvist et al., research covered younger groups of participants (pre- and just after maturation), while participants in our own studies were in the late phase of adolescence. Some results showed that girls change their BMI categories more often than boys with age [47]. Regardless of these differences, consistently with other studies, combined leisure time behaviors and intensity of after-school PA can strengthen the effect. [48]. This shows, on the one hand, the additive impact of positive or negative behaviors on body mass composition, and, on the other hand, justifies analyses based on multivariate statistical procedures as a more in-depth study of relationships [49]. Nonetheless, it is well-documented that more confounders support the effects of PA in poor body mass composition [50,51].

Our own results, consistently with others, indicated the importance of active commuting as a factor related to body mass composition. Many studies indicate that using active transport in work, school, or for private purposes constitutes an essential element of overall individual PA [52]. The great importance of active commuting may be related to the fact that adolescents must systematically, every day, spend some time getting to school or work. Hence, the large contribution of the active transport domain to the overall PA. Regular walking or cycling is a significant energy expenditure and contributes to the reduction in body fat [53]. Unfortunately, many studies demonstrated that declining rates of active transport is one of the consequences of, as well as a reason for, the growing inactivity in populations, and the evidence confirms that the increasing frequency of obese people is related to a decline in active transportation [54,55]. The current work showed active transport was the second most common source of PA volume (in overall PA MET-min/week) after leisure time. Its presence within the set of variables discriminating groups with different BFPs was not accidental. Systematic reviews demonstrated that active commuters are fitter and have a lower risk of obesity [56,57]. The results of the current study corroborate findings from other studies showing that walking or cycling to work is associated with lower body weight [58,59,60] and lower BFP [61].

A healthy diet is a foundation for health and well-being, while an unhealthy diet is a risk factor for various diseases [62]. The algorithm of discriminant analysis used combinations of the variables to search for the optimal set. Among the two DB indices, healthy and non-healthy, the importance of healthy behaviors in discriminating between the groups turned out to be greater. In this regard, more frequent healthy food consumption and eating unhealthy food less often were in competition. Nonetheless, eating healthy products more often was more specific and distinct when considered alongside the other three variables, which may explain the partial inconsistencies with previously noted results. A study on healthy and non-healthy dietary patterns suggested a crucial role for unhealthy DBs in obesity, and indicated that they were indirectly responsible for non-communicable diseases [63]. Furthermore, odds ratio (OR) data indicated that an unhealthy diet was more likely to increase overweight/obesity risk (OR = 1.65) compared to a prudent/healthy dietary pattern, which was more likely to lead to decreased body weight and fat mass (OR = 0.64) [64]. On the other hand, combining diet and PA improves weight loss more than every lifestyle component [65]. Indeed, those who changed their diet from unhealthy to healthy were 7.2 times more likely to lose weight than the control group. Moreover, improved dietary habits combined with increased PA enhanced the odds of losing weight (17.5-fold). Thus, these results are in agreement with the results presented in this work and confirm that healthy DBs are more vital than unhealthy behaviors when combined with other variables in a multidimensional set. Our own results are consistent also with findings in the Polish population suggesting a major role of healthy dietary behaviors combined with ‘Yard activity’ on BMI [9]. The findings showed, in addition, that participants with this kind of behavior were less likely to adhere to ‘Fast food and sweets’ dietary patterns. It is consistent with our previous findings [13]. Involvement in housework fills the free time of young people and can be a kind of protection against unfavorable factors (e.g., non-active sitting time or fast food or chips consumption) that affect body weight. The role of being involved in house chores was previously examined and results showed the associations with authoritative parenting style and dietary intake patterns [66].

A practical result of many studies is the set of regression equations to predict the value of dependent variables such as body weight components based on significant predictors. These equations can be used in various groups of people. Usually, different kinds of regression are used. In this article, the practical achievement was to determine the classification functions, which can be used to classify individuals based on a set of variables the most discriminated participants into 4 groups of BFP. Although our own methodology is quite different to regression analyses and classification equations cannot be directly compared or discussed with regression equations, our own results are consistent across identified variables. Taking into account the studied problem, the most often indicated predictors of overweight and obesity were the intensity of PA and healthy dietary behaviors (particularly consuming fruits and vegetables). Both domains were negatively related to body weight or body fat (the more PA or fruits and vegetables, the less body weight or fat) [46,67]. Moreover, infrequent physical activity was significantly associated with an almost 60% increased risk of being overweight or obese [46]. The same study showed that a low reported frequency of intake of vegetables was significantly associated with a 20% increased risk of being overweight or obese, and a low consumption of fish was associated with a 17% increased risk of being overweight or obese.

Limitations of this study include its cross-sectional design, which limited causal inference analysis. Another limitation is the inclusion of self-reported data. Despite empirical verification of the data reliability, the error resulting from such data acquisition is less robust than measuring directly. Moreover, collecting data through a web-based survey can affect accuracy. Nonetheless, a better response is expected from an online survey as respondents are more likely to complete it.

## 5. Conclusions

In conclusion, weak relationships between PA domains and DBs resulted from heterogeneous behaviors and mixed behavior patterns. Identification of the trajectory of the frequency flow through specific PA and DB areas allows for well-designed, tailored intervention programs that can promote healthy habits in adolescents. Thus, identifying the set of variables that most discriminate between lean, normal, and excessive fat groups is a reliable target for developing interventions. Intervention programs should use a combination of increased physical intensity, especially during leisure time, and healthy eating. It is also advisable to promote active transport on foot or by bicycle. Authorities and policy-makers could include a prescription for lowering body fat in education through a combined program of nutrition and PA, which could shape people’s awareness of the need to change their lifestyle habits. A practical achievement are the equations classifying the individuals based on a set of four variables (PA and DB variables) into four groups with different levels of body fat. These equations can be useful in conducting interventions.

## Figures and Tables

**Table 1:** Standardized mean values with 95% confidence intervals for variables measured in four groups of adolescents separated based on body fat percentage.

Standardized Variables	Lean			Lower Norm			Upper Norm			Excessive Fat		
	Mean	95% CI		Mean	95% CI		Mean	95% CI		Mean	95% CI	
body height	−0.12	−0.54	0.30	0.24	−0.16	0.64	−0.08	−0.40	0.24	−0.10	−0.53	0.34
body weight	−0.68	−0.95	−0.40	0.05	−0.28	0.38	−0.03	−0.40	0.34	0.61	0.17	1.06
body fat percentage	−0.84	−1.03	−0.65	−0.20	−0.46	0.07	−0.14	−0.41	0.14	1.20	0.85	1.55
work/school	−0.12	−0.56	0.33	0.12	−0.23	0.46	0.10	−0.28	0.49	−0.15	−0.57	0.28
active transport	0.01	−0.36	0.37	0.04	−0.36	0.44	−0.26	−0.60	0.08	0.23	−0.23	0.68
domestic/gardening	0.09	−0.33	0.51	−0.03	−0.37	0.32	−0.11	−0.57	0.34	0.07	−0.31	0.45
leisure time	0.31	−0.14	0.77	−0.43	−0.74	−0.12	0.17	−0.20	0.55	0.04	−0.38	0.47
vigorous	0.06	−0.43	0.56	−0.13	−0.47	0.22	0.07	−0.33	0.48	0.02	−0.34	0.38
moderate	−0.02	−0.43	0.38	0.01	−0.35	0.37	−0.15	−0.64	0.33	0.18	−0.14	0.50
walking	0.07	−0.31	0.46	−0.01	−0.40	0.37	0.03	−0.31	0.37	−0.09	−0.57	0.40
healthy diet	−0.06	−0.47	0.35	0.01	−0.39	0.40	−0.11	−0.50	0.28	0.16	−0.23	0.55
unhealthy diet	−0.28	−0.65	0.09	0.14	−0.26	0.54	0.00	−0.44	0.44	0.09	−0.26	0.44

**Table 2:** Wilk’s Lambda and tolerances for the effects derived from the discriminant analysis are presented for the best four subsets. The first subset contains the optimal set of variables for differentiating the four groups of participants.

Subset	Wilk’s Lambda	w/s	at	d/g	lt	Vig	Mod	Walk	Sit	Hdi	Unhdi
1	0.755		0.30 **		0.91 *			0.30 *		0.94	
2	0.755		0.30		0.93			0.30			0.99
3	0.763		0.28		0.59	0.62		0.30			
4	0.767	0.81	0.26		0.93			0.25			

w/s—work and school domain; at—active transport domain; d/g—domestic and gardening domain lt—leisure time domain; vig—vigorous intensity; mod—moderate intensity; walk—walking domain; sit—time spent sitting; hdi—healthy dietary behaviors; unhdi—unhealthy dietary behaviors; *—statistical significance *p* < 0.05; **—statistical significance *p* < 0.01.

**Table 3:** Raw and standardized (*β* in brackets) coefficients of 1st and 2nd discriminant function.

Function	Intercept	at	F (*p*)	lt	F (*p*)	Walk	F (*p*)	Hdi	F (*p*)
1	−0.12	−1.72 (−1.51)	5.49 (0.002)	0.51 (0.49)	3.86 (0.012)	1.59 (1.42)	5.07 (0.003)	−0.47 (−0.45)	2.17 (0.095)
2	0.10	0.64 (0.56)		0.94 (0.91)		−0.99 (−0.89)		−0.02 (−0.01)	

at—active transport domain; lt—leisure time domain; walk—walking domain; hdi—healthy dietary behaviors.

**Table 4:** The coefficients of the classification functions (calculated for each BFP group).

Function for Group	Intercept	at	lt	Walk	Hdi
lean	−1.80	−1.00	0.27	1.13	−0.59
norm 1	−1.39	−0.02	−0.58	0.21	0.08
norm 2	−1.52	−1.05	0.30	0.79	−0.01
excessive fat	−1.61	1.10	−0.01	−1.07	0.12

at—active transport domain; lt—leisure time domain; walk—walking domain; hdi –healthy dietary behaviors.

## Data Availability

The data presented in this study are available on request from the author.
